# Draft Genome Sequence and Brief History of Rhodovulum sp. Strain BSW8

**DOI:** 10.1128/MRA.00983-18

**Published:** 2018-08-23

**Authors:** Amiera A. Rayyan, Terry E. Meyer, John A. Kyndt

**Affiliations:** a College of Science and Technology, Bellevue University, Bellevue, Nebraska, USA; b Department of Chemistry and Biochemistry, University of Arizona, Tucson, Arizona, USA; Louisiana State University

## Abstract

Rhodovulum is a marine Gram-negative purple photosynthetic bacterial genus that is a member of the *Alphaproteobacteria*. Strain BSW8 is a variant that does not appear to make a polysaccharide slime capsule, and its genome sequence further contributes to the diversity of sequenced genomes belonging to this genus.

## ANNOUNCEMENT

The Rhodovulum genus was split from Rhodobacter based upon its salt requirement and marine habitat (1). We now know that these supposed differentiating characteristics are not unique to Rhodovulum species but also occur to a lesser extent in Rhodobacter species (2, 3). What is constant is the presence of a triheme cytochrome subunit in the photosynthetic reaction center in Rhodovulum species, which is absent in Rhodobacter species (4, 5). Elsden first isolated Rhodovulum species in van Niel’s laboratory and labeled them 2.7.1 to 2.11.1 but did not pursue them further (S. Elsden, personal communication). Hansen and Veldkamp isolated Rhodovulum sulfidophilum from marine mud flats (6), thereby proving that some nonsulfur purple bacteria not only tolerate but also utilize sulfide as an electron donor. R. sulfidophilum strain W4 was the first to have its genome sequenced (7–9). Unfortunately, R. sulfidophilum constitutively makes a polysaccharide capsule that interferes with isolation of cellular components (6, 10). Thus, Weaver isolated a close relative of R. sulfidophilum from the beach at the Scripps Institute of Oceanography (La Jolla, CA) that did not appear to make the slime capsule, which he labeled BSW8 (P. Weaver, personal communication). We found that the cytochromes of BSW8 were unlike those of R. sulfidophilum W4^T^ but were likely from a separate species, while the Elsden bacteria appeared to have the same cytochrome C_2_s as strain W4 (11). Since then, the genomes of Rhodovulum sp. strain MB263 (GenBank accession number NZ_CP020384), R. viride (GenBank accession number NZ_MUAV00000000), Rhodovulum sp. strain P5 (GenBank accession number NZ_CP015039), R. kholense (GenBank accession number NZ_QAYC00000000), and R. imhoffii (GenBank accession number NZ_QAAA00000000) have also been sequenced.

Frozen cells of Rhodovulum sp. strain BSW8 (from a purified culture isolated from a single colony) were transferred from Scripps Institute to the University of Arizona. The FastDNA spin kit for soil (MP Biomedicals) was used to isolate genomic DNA, and the DNA library was prepared using the Nextera DNA Flex library prep kit (Illumina). The genome was sequenced with an Illumina MiniSeq system using 500 μl of a 1.8 pM library. This yielded 668.97 Mbp of DNA from 2,215,122 paired-end reads (2 × 150-bp reads). Coverage was over 100×, which complicated assembly using Velvet version 1.2.10 (12). We therefore performed a random subsampling using the FastQ toolkit version 2.2.0 with a 50% sample read cutoff. The subsampled data set (1,107,561 reads) was assembled successfully *de novo* with Velvet. Velvet assembly used a minimum k-mer size of 21 and a maximum k-mer size of 121, and reverse complement reads were included. The assembled genome consisted of 82 contigs (>200 bp), with the largest contig having 388,164 bp and an *N*_50_ value of 124,485 bp. The genome sequence was annotated using Rapid Annotations using Subsystems Technology (RAST) version 2.0 (13). The BSW8 genome is 4.42 Mb; it has a GC content of 67.8% and 4,265 protein-encoding genes.

According to JSpecies average nucleotide identity (14), BSW8 is not particularly close to W4 but is 94% identical to R. kholense and R. viride, placing it on the borderline of a separate species. Both BSW8 and R. kholense are equidistant from R. sulfidophilum strain W4 at 86 to 87% identity, so they are clearly not R. sulfidophilum. R. viride and R. kholense are 98.0% identical to one another, while strain MB263 is an intermediate species between R. sulfidophilum W4 (92%) and BSW8 (87%). BSW8 is 75 to 78% similar to the remaining Rhodovulum species. The Rhodovulum species all have the *soxXYZABCDEF* thiosulfate utilization genes, and based on the genome sequences and the study by Hansen et al. (6), they all appear to tolerate and even utilize sulfide.

### Data availability.

This whole-genome shotgun project has been deposited at DDBJ/ENA/GenBank under the accession number QNVX00000000. The version described in this paper is version QNVX01000000. The raw sequencing reads have been submitted to the Sequence Read Archive (SRA), and the corresponding accession number is SRX4417069.
